# New institutionalisation following acute hospital admission: a retrospective cohort study

**DOI:** 10.1093/ageing/afw188

**Published:** 2016-10-15

**Authors:** Jennifer Kirsty Harrison, Azucena Garcia Garrido, Sarah J. Rhynas, Gemma Logan, Alasdair M. J. MacLullich, Juliet MacArthur, Susan Shenkin

**Affiliations:** 1 Alzheimer Scotland Dementia Research Centre, University of Edinburgh, Edinburgh, UK; 2 Centre for Cognitive Ageing and Cognitive Epidemiology, University of Edinburgh, Edinburgh, UK; 3 NHS Lothian, Edinburgh, UK; 4 Nursing Studies, University of Edinburgh, Edinburgh, UK; 5 Department of Clinical and Surgical Sciences, Geriatric Medicine, University of Edinburgh, Edinburgh, UK; 6 Corporate Nursing, NHS Lothian, Edinburgh, UK

**Keywords:** Institutionalisation, care home, cognition, interdisciplinary, standards, older people

## Abstract

**Background:**

institutionalisation following acute hospital admission is common and yet poorly described, with policy documents advising against this transition.

**Objective:**

to characterise the individuals admitted to a care home on discharge from an acute hospital admission and to describe their assessment.

**Design and setting:**

a retrospective cohort study of people admitted to a single large Scottish teaching hospital.

**Subjects:**

100 individuals admitted to the acute hospital from home and discharged to a care home.

**Methods:**

a single researcher extracted data from ward-based case notes.

**Results:**

people discharged to care homes were predominantly female (62%), widowed (52%) older adults (mean 83.6 years) who lived alone (67%). About 95% had a diagnosed cognitive disorder or evidence of cognitive impairment. One-third of cases of delirium were unrecognised. Hospital stays were long (median 78.5 days; range 14–231 days) and transfers between settings were common. Family request, dementia, mobility, falls risk and behavioural concerns were the commonest reasons for the decision to admit to a care home. About 55% were in the acute hospital when the decision for a care home was made and 44% of that group were discharged directly from the acute hospital.

**Conclusions:**

care home admission from hospital is common and yet there are no established standards to support best practice. Decisions should involve the whole multidisciplinary team in partnership with patients and families. Documentation of assessment in the case notes is variable. We advocate the development of interdisciplinary standards to support the assessment of this vulnerable and complex group of patients.

## Background

Care home admission, often termed institutionalisation, is a significant life event for an older person which is often portrayed with negativity [1]. In the UK, approximately 4% of the population over 65 years (~400,000 people) and around 20% of the population aged over 85 years reside in care homes [2]. The definition of care homes varies between countries: in the UK most places (e.g. 95% in Scotland) are occupied by long-stay residents, not expected to return to independent living in the community [3]. Although this transition may have negative practical and emotional impacts on the individual [4], it is often necessary for some older adults, particularly those with dementia [5].

In the UK, new institutionalisation occurs mostly through two routes: (i) community: from the patient's usual residence, or (ii) hospital: directly following an admission. Across England there is a sixfold variation in the likelihood of being transferred directly from the acute hospital into long-term care settings [6]. In Scotland, 47% of new long-term admissions come directly from hospital settings [3], although it is not known how many of these are from acute hospitals. Both NHS England and the Scottish Government policy argue that care home admission from acute hospitals should be avoided [7, 8]. In the community, transitions generally occur as a result of decisions made by the patient, their families and health and social care practitioners based on knowledge of the long-term health and functional status of the patient. Functional impairment, cognitive impairment and dementia are among the factors which predict institutionalisation [9, 10]. Studies have documented the process and experiences of those with dementia moving to a care home from community settings [11–13].

The processes involved in new institutionalisation following acute hospitalisation are poorly researched: we found no studies evaluating these. This is an important knowledge gap, given that >6,200 older adults experience such transitions every year in Scotland alone [3]. Several reasons can be suggested for a need for a higher level of care than can be provided in the community following acute hospital admission: the presenting medical problem [14] or worsening of existing chronic disease; the breakdown of social circumstances and support. Additionally, hospital admission can be associated with a decline in activities of daily living, functioning and independence [15, 16], potentially necessitating institutional care. Although these may be valid reasons for institutionalisation, some of these factors may be transient, and the considerable variability in rates of institutionalisation from hospital [6] suggest variability in decision-making processes and in turn this implies that some care home transitions may be unnecessary. Some have even argued that an admission to the acute hospital may lead to premature institutionalisation [17].

Our aim was to explore the patient characteristics, assessment processes and reasons involved in discharge to a care home following an acute hospital admission to a single large Scottish university hospital.

## Methods

We conducted a retrospective case note review of patients admitted from home to a large Scottish university hospital between November 2013 and February 2015 and discharged to a care home. Inclusion criteria were: adult patients (aged ≥18 years); admitted from a private residence; discharged to a care home without returning to their previous address. A consecutive series was sought and notes were accessed until *n* = 100 in the study cohort. We accessed notes at least 3 months after discharge to care home, incorporating those discharged from local specialist rehabilitation facilities. This was to allow time for the notes to be returned to medical records following discharge documentation being finalised.

Our sample was identified using the electronic patient management system (TRAK) using discharge destination codes. The patients’ TRAK electronic records and ward-based case notes were obtained and examined. The ward-based case notes included all medical, nursing and allied health professionals documentation and any documentation made on the ward by additional teams, such as social workers. The social work records are stored on a separate electronic social care system and were not included.

The Research Nurse (RN) evaluated all case notes against the study inclusion criteria. Cases were excluded if clinical coding incorrectly identified existing care home residents or individuals not discharged to a care home. Any patient re-admitted during the project data collection phase was excluded to allow clinical use of the case notes. These were grouped with other notes which could not be accessed for the research and classified as ‘unavailable records’. Finally, as our aim was to explore the assessment processes and discharge planning it was necessary for case notes to be complete, without periods of admission missing from the record. If significant gaps were identified without documentation, typically >1 week in duration, these were excluded and classified as ‘missing data’. This approach was discussed with the wider research team and agreed as appropriate. Exclusion was driven by incomplete data.

The research team (including consultant geriatricians, nurses, qualitative and quantitative researchers) developed and piloted a data collection form (Appendix 1). This was an iterative process to determine the variables to be included and the format for recording. The form was piloted by the RN and other members of the research team to support initial training and quality control. All data were extracted by a single RN onto the form, removing any identifiable data. A second researcher independently extracted data from a sample of case notes and their findings were compared with the RN to ensure consistency of approach.

### Cognitive status

We took an inclusive and pragmatic approach in classifying cognitive status and handling overlap. Detailed case defintions are included in Supplementary Data file (Appendix 2). If an individual received a new diagnosis of dementia during their stay, they were analysed in this group, even if they had a history of cognitive impairment. Delirium was assessed separately in view of the potential for this to fluctuate and improve.

### Ethics

The National Research Ethics Service approved the study (REC 14/44/1092), confirming that informed consent was not required from included participants. Caldicott Guardianship Approval (a UK system to protect patient information) [18] was granted.

### Statistical analysis

Two authors (JKH and AGG) entered data into IBM SPSS Statistics, version 21 (Release 21.0.0) and used this for analysis. Categorical variables were presented as frequencies and percentages with continuous variables presented as means with standard deviation (SD) or medians with ranges where data were not normally distributed.

## Results

### Cohort identification

To obtain a sample of 100 discharges, 273 case notes had to be evaluated. This resulted from incorrect coding (*n* = 44) of admission location or discharge destination, unavailability of case notes (*n* = 33; 9 case notes had been destroyed) and missing data (*n* = 96; 10 cases entire admission missing from notes).

### Description of the cohort

The sample (Table 1) were older adults (median 83.5 years; range 61–101), predominantly female (62%), living alone (67%), with a state-supported care package (73%). About 37% had a history of recurrent hospital admissions.

**Table 1.** Full description of the cohort

Variable	*N* (%)	Variable	*N* (%)
Female sex	62 (62)		
**Marital status**		**Family support**^**a**^	92 (92)
Widowed	46 (52)	Children/children-in-law	102 (71)
Married	26 (30)	Spouse/partner	21 (15)
Single	9 (10)	Niece/nephew	13 (9)
Divorced/separated	7 (8)	Other	7 (5)
Missing data	12		
**Cognitive diagnoses**		**Type of support provided**	
Cognitive impairment^b^	20 (42)	Shopping	62 (24)
Diagnosis of dementia AND/OR cognitive enhancers	55 (55)	Visiting/social	61 (23)
*Subtype recorded *(*n=52*)		Cleaning	54 (21)
Alzheimer's disease	17 (33)	Food preparation	40 (15)
Vascular dementia	12 (25)	Phone calls	22 (8)
Lewy body disease/Parkinson's disease-associated	4 (8)	Personal hygiene	11 (4)
Not specified	15 (29)	Dressing	9 (3)
		Other	4 (2)
		Missing data	13
**Past medical history**		**Frequency of support**	
Alcohol excess	10 (10)	Four times/day	5 (7)
Falls	63 (63)	Overnight	6 (8)
Depression	14 (14)	Daily	40 (57)
Recent previous admissions	37 (37)	Weekly	12 (17)
**Prescribing**		Monthly	3 (4)
Regular prescriptions	Median of 8 [range 0–21]	Missing data	32
Anti-psychotic use	17 (17)		
Cognitive enhancers	28 (28)		
**Functional status**		**State package of care**	73 (73)
**Mobility**		**Frequency**	
Zimmer frame	35 (35)	7 days/week	68 (93)
Stick	27 (27)	Four times/day	28 (38)
Unaided	27 (27)	Three times/day	15 (21)
Other	11 (11)	Twice daily	18 (25)
**Continence**		Once daily	9 (12)
Fully continent	57 (62)	Other	3 (4)
Incontinent of urine	31 (33)	**Nature of support**	
Doubly incontinent	4 (4)	Personal hygiene	60 (82)
Missing data	8	Medication prompting	47 (64)
**Use of continence aids**		Meal preparation	52 (71)
Continence pads	37 (40)		
Catheter	10 (11)		
**Social situation**		**Informal/unpaid support**	
**Living arrangements**		Friend	10 (16)
Lives alone	67 (67)	Neighbour	6 (10)
Lives with spouse/partner	25 (25)	Other	2 (3)
Lives with son/daughter	8 (8)	Missing data	39
**Housebound**	14 (16)		
**Missing data**	12		
**Type of property**		**Other formal services**	14 (25)
House	37 (42)	Day centre	7
Flat	24 (27)	Other (inc private carers)	7
Bungalow	13 (15)	Missing data	44
Sheltered accommodation	11 (12)		
Other	4 (4)		

Total sample is 100 therefore where data are complete, *n* = %. Where *n* is not equal to the % this represents data which were not available or not applicable.

^a^A total of 143 individuals were supporting 92 individuals; percentage represents proportion with at least one person in that category of relationship.

^b^Not possible to have diagnosis of dementia and cognitive impairment simultaneously so cognitive impairment result based on proportion without dementia.

### Circumstances of admission

All participants had an emergency rather than elective admission to hospital. Most were admitted under medicine (88%), with smaller numbers under orthopaedics (7%), surgery (4%) and joint care (1%). Admission reasons were recorded based on presenting complaints rather than diagnoses, so individuals often had multiple recorded reasons. Common reasons were: falls (57%); confusion (52%); sepsis (31%); neurological symptoms of stroke/TIA/seizure (14%); fractures (10%) and reduced mobility (8%).

### Cognitive disorders

The prevalence of all cognitive disorders was high (Table 2). Only five individuals had no formal diagnosis of cognitive impairment or evidence of impairment on cognitive testing. Formal cognitive assessment was attempted and recorded at least once in 77% of participants. The prevalence of cognitive impairment on formal testing was high (>80%), although detailed cognitive assessments were seldom used (Table 3).

**Table 2.** Cognitive test results

Cognitive test data	Abbreviated mental test score (AMT) *N* = 64	Mini-mental state examination (MMSE) *N* = 36	Addenbrooke's cognitive examination-III (ACE-III) *N* = 7
Mean [SD]	4.8 [3.8]	16.7 [5.4]	52 [11.6]
Median	5	16.5	56
Cut-off used	8 or lower	23 or lower	<82
Cognitive impairment (%)	53 (83)	31 (86)	7 (100)

25/64 who had an AMT 10 also had an MMSE and 5/64 had an ACE-III.3/36 who had an MMSE also had an AMT.

**Table 3.** Cognitive disorders

Cognitive disorders	No of cases/100
Dementia and cognitive Impairment	
Known dementia	56
Known cognitive impairment	16
New diagnosis of dementia or cognitive impairment	9
Undiagnosed cognitive impairment	9
No cognitive disorders	5
Not tested	5
Delirium	
Diagnosis of delirium	35
Undiagnosed delirium	16
No evidence of delirium	49

Cognitive impairment was identified in nine participants, without evidence of further diagnosis being made. Of these, two individuals had diagnosed delirium, two had evidence of delirium not formally diagnosed, two had challenging behaviour or depression with involvement of the hospital-based psychiatric liaison team, two cases where no follow-up or investigation was conducted and one diagnosed with depression.

About 28% were prescribed cognitive enhancers and 17% anti-psychotic medications. Of these, 18% were used in those who had co-morbid psychiatric history and 12% in those with behavioural and psychological symptoms of dementia or recognised ‘behavioural disturbance’ on admission.

### Events documented during admission

The median length of stay was 78.5 days (range: 14–231 days). Transfers of care were common, with 20% having one transfer, 47% having two transfers, 23% having three and 8% having more than three transfers during their admission and data missing for two individuals. Transfers were between parent wards, to rehabilitation settings, step-down care or boarding. About 50% of the cohort received off-site rehabilitation/complex discharge planning before discharge to care home and 45% experienced boarding.

#### Multidisciplinary team involvement

Physiotherapists saw 92% of the participants and their input was incorporated into the final discharge plan in 60 cases. Occupational therapists assessed 53% of participants and their input was incorporated into the final discharge plan in 42 cases. The hospital social work team was involved in 93% of cases.

#### Advance care planning

About 51% of the group had a Section 47 Adults with Incapacity (AwI) certificate and 10% had an advance statement. 45% had a recorded power of attorney or guardianship order of whom 53% had an AwI certificate. About 40% had a Do Not Attempt Cardio-Pulmonary Resuscitation order in their case notes.

#### Continence

Only 25/89 (28%) were documented as fully continent at time of hospital discharge (excluding those with no admission continence data), compared with 62% recorded as fully continent on admission.

#### Cognitive enhancers and anti-psychotic medications

About 4/28 (14%) had their cognitive enhancers stopped during admission and 4/72 (6%) were newly commenced on them. About 2/17 (12%) had their anti-psychotics stopped during admission and 13/83 (16%) were newly commenced on them.

### Discharge planning

In 42% of cases concerns about the individual's ability to cope living at home were raised on admission, 76% of these being raised by family members. Documentation of discharge planning was first discussed on a median of 6 days into admission (range 0–249). About 55% were in the acute hospital when the decision for a care home was made and 44% of them were discharged directly from the acute hospital.

The decision for care home admission was made on a median of 26 days after admission (range 0–249). The commonest main reason for the decision was family request (35%), followed by dementia (20%) and mobility limitations (9%). For five individuals, care home admission was at their own request.

## Discussion

Our cohort study of 100 patients provides the first characterisation of those admitted to a UK hospital and newly admitted to a care home: median age 83.6 years, mostly female with high levels of dependence, polypharmacy, incontinence and cognitive impairment. The overall picture is heterogeneous, with long hospital admissions, frequent transfers of care and varied levels of documented assessment.

This sample is representative of those admitted to care homes in the UK: prior participants in UK care home research are, on average age >80 years, majority female and with evidence of polypharmacy [19]. Most are widowed and lived alone, both identified as important predictors of need for institutional care in people with dementia [20, 21]. Levels of formal state-provided support were high (73%), reflecting the availability of free personal care for older adults in Scotland [22]. Recurrent hospitalisations, experienced by over a third of our cohort, are known to be associated with downward trajectories of disability among older adults [23] and can be an important indicator of increasing care needs.

All admissions were unscheduled and most were under medical specialties, following common presentations in an older adult population [24]. Length of stay is prolonged (mean 78 days) compared with the 2010 whole Scottish population average of 5.7 days [25]. Frequent transfers of care (31% moved ≥ three times and 45 experiencing boarding) have been associated with poorer outcomes for hospitalised older people [26]. However, some transfers were to allow access to rehabilitation in a non-acute hospital setting which is known to be associated with improved rates of independence [27].

Cognitive disorders—dementia [28] and delirium [29]—are known to be associated with an increased risk of institutionalisation. The prevalence of cognitive disorders in our sample was very high, only five individuals had no evidence of cognitive impairment. Delirium was unrecognised in one-third of cases. Clinicians must ensure discharge processes support those with cognitive disorders, including those lacking capacity. The use of case note review methodology allowed inclusion of people lacking capacity without burdening them or carers with a direct approach for participation in a research study.

Incontinence has previously been identified as a predictor of institutionalisation [30, 31]. We note that two-thirds were documented as fully continent on admission and less than a third were continent at discharge, and are exploring the reasons for this locally.

Functional status is an important predictor of care home admission following hospitalisation [32] and assessment should involve the multidisciplinary team (MDT). Just over half of our cohort had documented assessment by Occupational Therapy in contrast with more than 90% being assessed by Physiotherapy and hospital Social Work. It is likely that functional needs were discussed in MDT meetings and detailed assessment (beyond observations of experienced nurses) not felt to be appropriate, but this was not recorded in the case record. The latest National Institute for Health and Care Excellence guideline about transitions of older people between health and social care [33] recommends comprehensive assessment for older people with complex needs by an MDT, although it is flexible on who should be involved in the team [33]. High quality specialist assessment is advantageous for an individual's ongoing management within a care home setting [34], provided such information is shared [6].

Concerns about the individual's ability to manage at home were frequently raised on admission to hospital and discharge planning was discussed early, in keeping with current policy approaches in favour of early discharge planning [35]. The formal decision for care home admission was appropriately later in the admission for the majority.

Primary reasons for care home admission concur with known predictive factors of care home admission from the community [10, 36, 37].

The main limitation is that the information was extracted from ward-based case notes. Undocumented conversations could not be included and we have no way to determine the frequency or scale of this issue. We included information entered in the notes by social workers, but could not included the detailed social assessments which are entered by social workers on a separate electronic system. Increased use of electronic health records and integration of health and social care should help to improve this. Some important clinical information, such as cognitive function and mobility at discharge, is also not routinely re-assessed or recorded.

Another limitation relates to the sampling approach. We sought a consecutive sample of discharges, however coding inaccuracy, unavailable records and missing data required us to screen 273 case notes to obtain 100. Identifying coding inaccuracy is important to ensure the integrity of our study. We are sure all of our sample were discharged to a care home, having previously resided in a non-institutional setting. However, this highlights the potential for the use of routine hospital data to misclassify residency status. Exclusion of cases based on missing data and unavailable records introduces the potential for selection bias. However, this potential bias has to be balanced against our aim which was to evaluate the assessment processes and decision. This is a pragmatic health services research study and for the findings to have validity, this evaluation cannot be made with confidence if records are incomplete, missing completely or destroyed. Paper-based records are susceptible to missing data and cannot be simultaneously used for detailed case note research and clinical care. If the reasons for missing data and unavailable records were non-random, this would result in a biased sample. The results for this cohort are, however, consistent with previous findings and informative.

Data extraction was performed by a single researcher and thus creates the potential to introduce bias. However, the data extraction tool was developed and piloted with a wider group of clinicians and researchers. Furthermore, data extraction was not based on any prespecified hypotheses so there is no reason to predict bias in one particular direction.

We collected data at a single centre, so the issues may not be more widely applicable. These data allow practitioners to review their own local service and processes for this common clinical scenario. The absence of primary research in this area is striking and further research is needed to define best practice in care home decision-making.

We did not perform a case control study, and are unable to compare the experiences of our cohort with those who were not discharged to a care home. Our sample also has a ‘survivor bias’ as only those who lived through their hospital admission to be discharged were included.

Finally, the voice of individual patients is not heard through the assessment of ward-based case notes and this is problematic in evaluating decision-making. We recognise the complexity and individuality of each person being admitted to a care home, but suggest that the lack of established formal standards of care and variations in national practice must raise concern.

To ensure the right decision is made with each individual, we would advocate the development of standards in the assessment of an older person in hospital being discharged to a care home. These would provide a framework to support the individual, the family and the hospital team in making this life-changing decision.

Key points

- Care home admission from hospital is an under-researched, but common occurrence in the UK.
- Cognitive impairment is highly prevalent among this population.
- Variation in assessment and practice is evident on case note review.
- We advocate the development of interdisciplinary standards to support this life-changing process.

## Supplementary Material

Supplementary Data

Supplementary Data
