# Supplementary material for: New institutionalisation following acute hospital admission: a retrospective cohort study
Source: Age Ageing. 2016 Oct 15;46(2):238–44. doi: 10.1093/ageing/afw188 (PMC5860512; doi:10.1093/ageing/afw188)
Supplement: Supplementary Data [file aa-16-0435-file001.pdf]

EDDAC Data Extraction Form **Unique Identification Number……………………..**
Version 4
2nd April 2015

**Section A**	
**1. Date of Admission**	
**2. Age**	Years………
**3. Sex**	Male ☐ Female ☐
**Section B**<br>**Pre-Admission Circumstances**	
**4. Past Medical History/Existing Diagnoses (Tick all that apply)**	Episodes of Delirium ☐ Confusion ☐<br>Cognitive Impairment ☐ Alcohol excess ☐<br>Dementia (specify type below) ☐<br>Vascular ☐ Alzheimer's Disease ☐<br>Type not specified ☐ None of the above ☐<br>Other (please specify) ☐ …................................
**5. Associated Anti-Dementia medication prior to admission**	Donepezil (Aricept) ☐<br>Galantamine (Reminyl) ☐<br>Rivastimine (Exelon) ☐<br>Memantine (Ebixa) ☐<br>None ☐
**6. Associated Anti-Psychotic medication prior to admission**	Haloperidol (Dozic/Haldol/Serenace) ☐<br>Quetiapine (Seroquel) ☐<br>Trazadone (Molipaxin) ☐<br>Risperidone (Risperdal) ☐<br>Chlorpromazine ☐<br>None ☐ Other (please specify) ☐<br>…...
**7. Total number of regular prescriptions (exclude vitamins, inhalers, over the counter medications)**	………………………………………………………….<br>.
**8. Mobility prior to admission**	Walks unaided ☐ Walks with a stick ☐<br>Walks with a zimmer frame ☐<br>Other (please specify) ☐<br>………………………………………………………….
**9. Did the patient have a history of falls (prior to admission)?**	Yes ☐ No ☐ Not documented ☐

**10. Continence (Tick all that apply)**	Fully continent ☐ Incontinent of Urine ☐ Doubly-Incontinent ☐ Not documented ☐
**11. Did the patient have continence aids?**	Continence Pads ☐ Catheterised ☐ Uro-sheath ☐ None required ☐
**Section C Social Circumstances**	
**12. Living Arrangements**	Lives alone ☐ Lives with partner ☐ Lives with husband/wife ☐ Lives with son ☐ Lives with daughter ☐ Not documented ☐ Other (please specify) ☐ ………………………………………………………………
**13. Housebound**	Yes ☐ No ☐ Not documented ☐
**14. Type of Property**	House ☐ Sheltered Housing ☐ Flat ☐ Bungalow ☐ Not documented ☐ Other (please specify) ☐ ………………………………………………………………
**15. Stairs within property**	Yes ☐ No ☐ Not documented ☐
**16. Stairs leading to property**	Yes ☐ No ☐ Not documented ☐
**17. Marital Status**	Married ☐ Civil Partnership ☐ Single ☐ Divorced ☐ Widowed ☐ Separated ☐ Not documented ☐
**18. What family members does the patient receive support from? (Tick all that apply)**	Husband/Wife/Partner ☐ Son ☐ Son-in-law ☐ Daughter ☐ Daughter in-Law ☐ Niece ☐

	Nephew ☐ No family support ☐<br>Not documented ☐<br>Other (please specify) ☐<br>……………………………………………………………
**19. What type of support do they receive from family members? (Tick all that apply)**	Dressing ☐ Personal hygiene ☐<br>Food Preparation ☐ Visiting/Social ☐<br>Cleaning ☐ Shopping ☐<br>Telephone Calls ☐ No family support ☐<br>Not documented ☐<br>Other (please specify) ☐<br>……………………………………………………………
**20. How frequently is the support given by the family member? (Tick all that apply)**	Daily ☐ Alternate days ☐<br>Weekly ☐ Alternate weeks ☐<br>Monthly ☐ Less than once a month ☐<br>1 x per day ☐ 2 x per day ☐<br>3 x per day ☐ 4 x per day ☐<br>Overnight ☐ Not documented ☐<br>Not Applicable ☐
**21. What type of informal/unpaid social support does the patient receive (non-family members and not including POC) (Tick all that apply)**	Friend ☐ Neighbour ☐<br>No social support ☐ Not documented ☐<br>Other (please specify) ☐ …………………………………
**22. How frequently is the informal/unpaid support received? (Tick all that apply)**	Daily ☐ Alternate days ☐<br>Weekly ☐ Alternate weeks ☐<br>Monthly ☐ Less than once a month ☐<br>1 x per day ☐ 2 x per day ☐<br>3 x per day ☐ 4 x per day ☐<br>Overnight ☐ Not documented ☐<br>Not Applicable ☐

**23. Does the patient receive support from any formal services? (Do not include POC) (Tick all that apply)**	Day centre ☐ Day Hospital ☐<br>Not received ☐ Not documented ☐<br>Other (please specify) ☐<br>………………………………………………………
**24. If the patient attended a day centre how many days per week do they attend?**	1 ☐ 2 ☐ 3 ☐ 4 ☐<br>5 ☐ Not Applicable ☐
**25. Does the patient attend any additional support groups?**	Yes (please specify) ☐ No ☐<br>Not documented ☐<br>Support group ………………………………………
**26. If the patient attends any additional support groups, how many days per week do they attend?**	1 ☐ 2 ☐ 3 ☐ 4 ☐<br>5 ☐ 6 ☐ 7 ☐<br>Not documented ☐ Not Applicable ☐
**Section D**<br>**Pre-admission Package of Care:**	
**27. Was the Patient in receipt of a package of care?**	Yes ☐ No ☐ Not documented ☐
**28. If yes, how many days per week is this received?**	1 ☐ 2 ☐ 3 ☐ 4 ☐<br>5 ☐ 6 ☐ 7 ☐ Not App. ☐
**29. Frequency of visit**	1 x per day ☐ 2 x per day ☐<br>3 x per day ☐ 4 x per day ☐<br>More than 4 x visits ☐ Overnight ☐<br>Not Applicable ☐ Not documented ☐
**30. What type of care/assistance was provided? (Tick all that apply)**	Personal Hygiene/Dressing ☐<br>Medication Prompting ☐<br>Meal Preparation ☐<br>Not documented ☐ Not Applicable ☐
**31. Did the patient have a power of attorney/guardian?**	Yes ☐ No ☐ Not documented ☐
**32. Did the patient have an Advance Statement**	Yes (provide details) ☐ No ☐<br>Not documented ☐<br>Details………………………………………………<br>………………………………………………………

**33. Is there an order under the Adults with Incapacity Act?**	Yes (provide details) ☐ No ☐<br>Not documented ☐<br>Details………………………………………………….<br>………………………………………………………....
**Section E**<br>**Hospital Admission Circumstances**	
**34. Type of Admission**	Emergency ☐ Elective ☐
**35. Speciality on admission**	Medical ☐ Surgical ☐ Orthopaedic ☐
**36. If medical (including MOE), specify reason**<br>**(Tick all that apply)**	Confusion ☐ Falls ☐<br>UTI ☐ Pneumonia ☐<br>COPD ☐ Not medical ☐<br>Other (please specify)<br>………………………………………………………….
**37. If surgical, specify reason**<br>**(Tick all that apply)**	Gallstones ☐ Diverticulitis ☐<br>Cardiac ☐ Not surgical ☐<br>Other (please specify) ☐<br>………………………………………………………….
**38. If orthopaedic, specify reason**<br>**(Tick all that apply)**	Hip fracture ☐ Not orthopaedic ☐<br>Other (please specify) ☐<br>………………………………………………………..
**39. Were concerns regarding ability to cope at home raised on admission?**	Yes (provide details) ☐ No ☐<br>By whom? …..
**40. Total length of stay in hospital (days)**	
**41. Total length until "boarding" (delayed) (days)**	….. Not delayed ☐ Not documented ☐
**42. Total number of transfers**	1 ☐ 2 ☐ 3 ☐ 4 ☐<br>5 ☐ 6 ☐ 7 ☐ 0 ☐
**43. List all areas transferred to**<br>**(Tick all that apply)**	Parent Ward (MOE/Orth./Surg.) ☐<br>Rehabilitation ☐ Boarding ☐<br>Step Down ☐ Not transferred ☐

	Not documented ☐ Other (please specify) ☐<br>……………………………………………………………....
**Section F**<br>**Inpatient Circumstances**	
**44. Stratified Falls Risk Assessment score on admission**	1 ☐ 2 ☐ 3 ☐ 4 ☐<br>5 ☐ Not documented ☐
**45. Did this score change during inpatient stay?**	Yes (provide date) ☐ No ☐<br>Not documented ☐<br>Date(s) ………………………………………………....
**46. Final score**	1 ☐ 2 ☐ 3 ☐ 4 ☐<br>5 ☐ Not Applicable ☐
**47. Total number of falls during inpatient stay**	………………………………………………………………<br>.
**48. Provide details of cognitive assessment undertaken during inpatient stay**	No cognitive test undertaken ☐<br>4AT ☐<br>Date(s) and score<br>……………………………………………………………………<br>AMT ☐<br>Date(s) and score<br>………………………………………………………………<br>MMSE ☐<br>Date(s) and score<br>………………………………………………………………<br>ACE3 ☐<br>Date(s) and score<br>……………………………………………………………
**49. Was the patient referred for specific mental health support during their inpatient stay (e.g. Bridging Team, Behavioural Support)?**	Yes (provide details) ☐ No ☐<br>Date……………………………………………………......<br>Support Team……………………………………………..<br>Reason…………………………………………………….

**50. Was there evidence of delirium during inpatient stay?**	Yes (specify date/s) ☐ No ☐<br>Date/s…………………………………………………....
**51. If yes, provide details of the cause, duration and treatment.**	Cause………………………………………………..<br>Duration……………………………………………….<br>Treatment…………………………………………...<br>……………………………………………………......<br>Not Applicable ☐
**52. Diagnoses made during inpatient stay**	Cognitive impairment ☐<br>Date……………………………………………….....<br>Dementia ☐<br>Date……………………………………………….....<br>Delirium ☐<br>Date…………………………………………………...<br>Confusion (no details provided) ☐<br>Date …...<br>None ☐
**53. Was there any evidence of agitation during inpatient stay?**	Yes (specify details) ☐ No ☐<br>Date/s………………………………………………....<br>Details………………………………………………..<br>………………………………………………………….
**54. Was there any evidence of aggression during inpatient stay?**	Yes (specify details) ☐ No ☐<br>Date/s………………………………………………....<br>Details…………………………………………………<br>………………………………………………………….
**55. Was there any evidence of absconding during inpatient stay?**	Yes (specify details) ☐ No ☐<br>Date/s………………………………………………....<br>Details…………………………………………………<br>………………………………………………………....

**56. Was there any evidence of the use of a Wander-guard during inpatient stay?**	Yes (specify details) ☐ No ☐<br>Date/s…………………………………………………....<br>Details…………………………………………………<br>…………………………………………………………
**57. Landmark Occupational Therapy Assessment(s)**	Yes (please specify) ☐ No ☐<br>Personal care ☐<br>Date(s)<br>……………………………………………………<br>Kitchen ☐<br>Date(s)<br>……………………………………………………<br>Home visit ☐<br>Date(s)<br>……………………………………………………<br>Other (please specify) ☐<br>Assessment……………………………………………..<br>Date(s)<br>………………………………………………………........
**58. Were the OT contributions incorporated into the final discharge plan?**	Yes ☐ No ☐ Not documented ☐
**59. Landmark Physio Assessment(s)**	Yes (please specify) ☐ No ☐<br>Type of assessment…………………………………….<br>Frequency: Only on admission ☐<br>Daily ☐ Weekly ☐<br>Other assessments<br>…..........................……..<br>Date(s)<br>…..
**60. Were the Physio contributions incorporated into the final discharge plan?**	Yes ☐ No ☐ No documented ☐
**61. Was the patient referred to Social Work during inpatient stay?**	Yes (provide date) ☐ No ☐<br>Date……………………………………………………......

**62. During the inpatient stay were there concerns raised about the patient's ability to cope/manage at home?**	Yes (provide initial date) ☐ No ☐<br>Date………………………………………………………...
**63. If yes, who was this instigated by?**	Patient ☐ Relative ☐ Friend ☐<br>Neighbour ☐ Nurse ☐ OT ☐<br>Physio ☐ Medical Staff ☐ Not Applicable ☐<br>Other (please specify) ☐<br>………………………………………………………………
**64. During the inpatient stay detail evidence of ongoing liaison with family**	Regular ☐ Infrequent ☐<br>Recorded ☐ No evidence ☐
**Section G<br>Discharge Planning**	
**65. On what date was discharge first discussed?**	………………………………………………………………<br>Not documented ☐
**66. What was the expected destination at this stage?**	Previous living arrangements ☐<br>Care home ☐<br>New living arrangements (not care home) ☐<br>Specify…………………………………………………......<br>Not documented ☐
**67. At this stage if the expected destination was NOT a care home, provide details relating to package of care expectations**	No POC ☐ Existing POC ☐<br>Increased POC ☐ Not Applicable ☐<br>Not documented ☐
**68. Was the decision for the patient discharge to a care home clearly documented?**	Yes ☐ No ☐
**69. In what area was the decision to discharge to care home made?**	Parent ward ☐ Boarding ☐<br>Rehabilitation ☐ Step Down ☐<br>Not documented ☐ Not transferred ☐<br>Other (please specify) ☐<br>………………………………………………………………
**70. Provide date of when the decision to discharge to care home was made**	………………………………………………………………

**71. Who was involved in the decision to discharge the patient to a care home? (Tick all that apply)**	Patient ☐ Family member ☐<br>MDT ☐ Not documented ☐
**72. Were members of the MDT in agreement with the decision to discharge the patient to a care home?**	Yes ☐ No ☐ Not documented ☐<br>Not possible to tell ☐
**73. If members of the MDT were NOT in agreement with the decision to discharge the patient to a care home, what was the reason?**	Believed patient was coping ☐<br>Believed patient would cope with increased support ☐<br>Not applicable ☐ Other (please specify) ☐<br>………………………………………………………………
**74. What was the MAIN reason for discharge to a care home? (Tick one only)**	Patient choice ☐ Family request ☐<br>Dementia ☐ Patient wandering ☐<br>Mobility ☐ Falls risk ☐<br>Nutritional risk ☐ Patient lacking confidence ☐<br>Behavioural concerns ☐ Isolation/loneliness ☐
**75. Provide details of any other factors that were documented for reason for discharge to a care home. These may be reasons that contributed to the decision, but were not the MAIN reason. (Tick all that apply)**	Patient choice ☐ Family request ☐<br>Dementia ☐ Patient wandering ☐<br>Mobility ☐ Falls risk ☐<br>Nutritional risk ☐ Patient lacking Confidence ☐<br>Behavioural concerns ☐ Isolation/loneliness ☐<br>Not possible to tell ☐ No other reason documented ☐<br>Other (please specify) ☐<br>………………………………………………………………
**76. Anti-dementia medications at discharge**	Donepezil (Aricept) ☐<br>Galantamine (Reminyl) ☐<br>Rivastimine (Exelon) ☐<br>Memantine (Ebixa) ☐<br>None ☐

**77. Anti-psychotic medications at discharge**	Haloperidol (Dozic/Haldol/Serenace) ☐<br>Quetiapine (Seroquel) ☐<br>Trazadone (Molipaxin) ☐<br>Risperidone (Risperdal) ☐<br>Chlorpromazine ☐<br>None ☐
**78. Continence at discharge**	Continent ☐ Catheter ☐<br>Incontinent with aids (e.g. pads) ☐
**79. Discharge Date**	…………………………………………………………………
**80. Additional Notes and Overall Impression**	…………………………………………………………………<br>.<br>…………………………………………………………………<br>.<br>…………………………………………………………………<br>.<br>…………………………………………………………………<br>.<br>…………………………………………………………………<br>.<br>…………………………………………………………………<br>.<br>…………………………………………………………………<br>.<br>…………………………………………………………………<br>.<br>…………………………………………………………………<br>.<br>…………………………………………………………………<br>.<br>…………………………………………………………………<br>.<br>…………………………………………………………………

Date of completion **Signature of person completing the form**
