# Supplementary material for: New institutionalisation following acute hospital admission: a retrospective cohort study
Source: Age Ageing. 2016 Oct 15;46(2):238–44. doi: 10.1093/ageing/afw188 (PMC5860512; doi:10.1093/ageing/afw188)
Supplement: Supplementary Data [file aa-16-0435-file002.docx]

Suppelemtary Data

[Please insert PDF file “[Appendix 1 Data collection sheet.pdf (v1.0)](https://mc.manuscriptcentral.com/LongRequest/ageing?DOWNLOAD=TRUE&PARAMS=xik_Rp9xqAdRyviHR9yCBmjJi3u5Rk2A3ueGhqRH9K4w2GCkept2QpejEXaVmw1o85Y6ooSWJ5MAHMRv7s5iPM5dJLq4tL4d1GS6AkU1tQbSLzRPSxABzBP5vfyRXW5MJYXP7jyrw5GJKfzVeWPmJXj2dhKMy77WTqNFMkBBCHSWYRWpsCw)” for Appendix 1]

*Appendix 2: Case definitions*

Dementia & cognitive impairment:

1. Known dementia –past medical history of dementia and/or an admission prescription for cognitive enhancing medication
2. Known cognitive impairment – past medical history of cognitive impairment
3. New diagnosis of dementia or cognitive impairment – cases where individuals received a diagnosis of either dementia or cognitive impairment, excluding those with a past medical history diagnosis of same
4. Undiagnosed cognitive impairment – using cognitive test scores (see below)
5. No chronic cognitive impairment – those with no history of dementia or cognitive impairment, no new diagnosis of dementia or cognitive impairment and no evidence of impairment on cognitive testing

**Undiagnosed Cognitive Impairment**

Data were recorded whenever a cognitive test was performed, including the score and date. The best (i.e. highest) score was used when tests were repeated. The tools used were: The Abbreviated Mental Test,[1] the 4AT a rapid assessment for delirium and cognitive impairment, The Mini-Mental State Examination (MMSE)[2] and Addenbrooke’s Cognitive Examination (ACE-III).[3] Established cut-off values to define cognitive impairment were used: AMT (eight or lower);[4] MMSE 23 or lower[5] and ACE-III <82.[6]

Delirium:

1. Episode of diagnosed delirium – where a diagnosis of delirium was made in the case record at any point during admission
2. Evidence of delirium without diagnosis – evidence suggestive of delirium on case note review
3. No delirium – no diagnosis of delirium and no evidence of undiagnosed delirium

**Undiagnosed Delirium**

Our research assistant (an experienced nurse in geriatric medicine) reviewed all of the case notes to complete data extraction. Undiagnosed delirium was recorded based on clinical judgement of case scenarios using the medical and nursing records, e.g. cases of ‘acute confusion’, ‘atypical behaviour disturbance’ etc. were included if delirium was thought to be likely albeit not labelled as such by the care team.

References

1. Hodkinson HM. Evaluation of a mental test score for assessment of mental impairment in the elderly. Age Ageing. 1972 Nov;1(4):233-8.

2. Folstein M, Folstein S, McHugh P. "Mini-mental state". A practical method for grading the cognitive state of patients for the clinician. Journal of Psychiatric Research. 1975;12(3):189-98.

3. Addenbrooke's Cognitive Examination - ACE-III (English Version A). 2012 [cited 2015 13th October]; Available from: <http://dementia.ie/images/uploads/site-images/ACE-III_Administration_(UK).pdf>.

4. Jitapunkul S, Pillay I, Ebrahim S. The abbreviated mental test: its use and validity. Age Ageing. 1991 Sep;20(5):332-6.

5. Tombaugh TN, McIntyre NJ. The mini-mental state examination: a comprehensive review. J Am Geriatr Soc. 1992 Sep;40(9):922-35.

6. Hsieh S, Schubert S, Hoon C, Mioshi E, Hodges JR. Validation of the Addenbrooke's Cognitive Examination III in frontotemporal dementia and Alzheimer's disease. Dement Geriatr Cogn Disord. 2013;36(3-4):242-50.
